# Supplementary material for: From single decisions to sequential choice patterns: Extending the dynamics of value-based decision-making
Source: PLoS One. 2022 Apr 21;17(4):e0267249. doi: 10.1371/journal.pone.0267249 (PMC9022817; doi:10.1371/journal.pone.0267249)
Supplement: S1 Appendix — (DOCX) [file pone.0267249.s001.docx]

From single decisions to sequential choice patterns: Extending the dynamics of value-based decision-making

# S1 Appendix. Computational Modelling and Simulation

Stefan Scherbaum^1*,^ Steven J. Lade^2,3,4^, Stefan Siegmund^1^, Thomas Goschke^1^, Maja Dshemuchadse^5^

^1^Technische Universität Dresden, Dresden, Germany

^2^Max Planck Institute for the Physics of Complex Systems, Dresden, Germany

^3^Stockholm University, Stockholm, Sweden

^4^The Australian National University, Canberra, Australia

^5^Hochschule Zittau-Görlitz, Görlitz, Germany

* Corresponding author

Stefan Scherbaum

Department of Psychology, Technische Universität Dresden

Zellescher Weg 17, 01062 Dresden, Germany

Phone: ++49 351 463 33258

E-mail: Stefan.Scherbaum@tu-dresden.de

*Keywords:* Decision-making, time-scales, sequential patterns, attractor model

The formalized model used for the simulations below built on models which have been used to study the neural activation dynamics of perceptual decisions in the past [compare 1–3]. Recently, we showed that it can also be applied to delay discounting, where it successfully predicted within and across trial effects [4,5].

These two units inhibit each other which opens a natural mapping of these two units to the architecture of more complex models, i.e. parallel constraint satisfaction networks [e.g. 6]. The two units in the model here could be seen as the two options in a parallel constraint satisfaction network that are fed by informative cues from lower network levels. By interactive activation and competition both systems lead to a settled decision state in the end of the decision process.

## Mathematical model description

The dynamics of the model are defined by two coupled differential equations (one for each neural unit) representing non-linear neural activation dynamics [1,2,see also 7,8].

$$\tau{\overset{\cdot}{u}}_{SN}=-u_{SN}+h+w_{r}\cdot\sigma\left( u_{SN} \right)+w_{i}\cdot\sigma\left( u_{LF} \right)+I_{SN}+q\xi_{SN}\left( t \right)$$

$$\tau{\overset{\cdot}{u}}_{LF}=-u_{LF}+h+w_{r}\cdot\sigma\left( u_{LF} \right)+w_{i}\cdot\sigma\left( u_{SN} \right)+I_{LF}+q\xi_{LF}\left( t \right)$$

Here, *τ* denotes the timescale (defining the step size of the Euler solution), *h* denotes the resting level, *w_i_* the (inhibitory) coupling strength of the two equations, and w*_r_* the recurrent feedback, *I_SN_* and *I_LF_* the input representing the attractiveness of the two options, *q* the strength of the normally distributed noise ξ, and *σ* a sigmoid non-linearity, mirroring non-linear neural population dynamics:

$$\sigma\left( u \right)=\frac{1}{\left( 1+e^{-\beta u} \right)}$$

Hence, interactions between the two units only happen to the extent that the activation *u* exceeds a soft threshold [9,10]. Note that the *β*-parameter is also called the gain-parameter, modulating the discreteness of neural activation states, which we varied across simulated subjects [for hypotheses concerning β and aging, see e.g. ,11]. For a list of the chosen parameter values, see Table 1. Importantly, these parameters are identical to the ones from our recent applications of this model to delay discounting [4,5] with exception of the strength *q* of the added normally distributed noise (the previous versions ran without any noise) and the random variation of *β* across simulated subjects (the previous versions ran only one prototypical subject).

Table 1. Basic parameters of the model

| Parameter | Value |
| --- | --- |
| τ | 10 |
| h | -5 |
| w_i_ | -7 |
| w_r_ | 6 |
| q | 0.1 |
| β | 1 ± 0.2 std across 20 simulated subjects |

The dynamics of the system are governed by the inputs to the two units, that is *I_SN_* and *I_LF_* To drive the units near to the activation threshold, these inputs consisted of a base Input *I_base_* modified by the relative attractiveness of the two options

$$I=I_{base}\pm I_{attractiveness}$$

As in the recent application of the model *I_base_* was set to 6. *I_attractiveness_* was set to 0.05, yielding e.g. for the condition of an attractive LF option *I_SN_* = 5.95 and *I_LF_* = 6.05.

## Simulation design

We simulated three conditions, an attractive SN option, a neutral condition, and an attractive LF option by varying *I_attractiveness_* in the described way. We simulated sequences of trials within each condition, with each sequence consisting of 30 consecutive trials and replicated 4 times, leading to an overall number of 360 simulated trials (3 conditions x 4 replications x 30 trials). With this setup, we simulated 20 subjects, varying the gain parameter *β* between subjects by a standard deviation of 0.2.

## Simulation procedure

The two coupled differential equations constitute a neural system with two units inhibiting each other so that only one unit can win the competition and elicit the final choice. This competition unfolds over time. We simulated the behaviour of the system by numerical integration (using the Euler method with the step size τ as described above) with each trial having a maximum length of 200 time steps (considering average simulated response times below 30 circles, this offers enough time for all trials to find a decision). Results were obtained using Matlab 2010a running on a Windows 8 platform.

In each trial, we presented to the network at time *t* = 60 the two options. A choice was elicited when one unit’s output reached a threshold of 0.85. The simulation was run continuously from trial to trial, so that the intrinsic dynamics of the system could lead to the predicted perseveration phenomena.

To simulate the influence of ITI length on perseveration, we reran the original simulations with two different ITI lengths, one time presenting the options at *t* = 55 and one time at *t* = 65.

# References

1. Hock HS, Schöner G, Giese M. 2003 The dynamical foundations of motion pattern formation: Stability, selective adaptation, and perceptual continuity. *Percept. Psychophys.* **65**, 429–457.

2. Noest AJ, van Ee R, Nijs MM, Wezel RJA van. 2007 Percept-choice sequences driven by interrupted ambiguous stimuli: A low-level neural model. *J. Vis.* **7**, 10.

3. Usher M, McClelland JL. 2001 The time course of perceptual choice: The leaky, competing accumulator model. *Psychol. Rev.* **108**, 550–592.

4. Scherbaum S, Frisch S, Leiberg S, Lade SJ, Goschke T, Dshemuchadse M. 2016 Process dynamics in delay discounting decisions: An attractor dynamics approach. *Judgement Decis. Mak.* **11**, 472–495.

5. Scherbaum S, Frisch S, Dshemuchadse M. 2018 Step by step: Harvesting the dynamics of delay discounting decisions. *Q. J. Exp. Psychol. 2006* **71**, 949–964. (doi:10.1080/17470218.2017.1307863)

6. Glöckner A, Betsch T. 2008 Modelling option and strategy choices with connectionist networks: Towards an integrative model of automatic and deliberate decision making. *Judgm. Decis. Mak.* **3**, 215–228.

7. Amari S. 1977 Dynamics of pattern formation in lateral-inhibition type neural fields. *Biol. Cybern.* **27**, 77–87.

8. Wilson HR, Cowan JD. 1972 Excitatory and Inhibitory Interactions in Localized Populations of Model Neurons. *Biophys. J.* **12**, 1–24.

9. Cohen JD, Servan-Schreiber D, McClelland JL. 1992 A parallel distributed processing approach to automaticity. *Am. J. Psychol.* **105**, 239–269.

10. Erlhagen W, Schöner G. 2002 Dynamic field theory of movement preparation. *Psychol. Rev.* **109**, 545–572.

11. Li S-C, Lindenberger U, Sikström S. 2001 Aging cognition: from neuromodulation to representation. *Trends Cogn. Sci.* **5**, 479–486.
